# Alcohol Harm Reduction: Corporate Capture of a Key Concept

**DOI:** 10.1371/journal.pmed.1001767

**Published:** 2014-12-09

**Authors:** Jim McCambridge, Kypros Kypri, Colin Drummond, John Strang

**Affiliations:** 1 Faculty of Public Health and Policy, London School of Hygiene & Tropical Medicine, London, United Kingdom; 2 Centre for Clinical Epidemiology and Biostatistics, School of Medicine and Public Health, University of Newcastle, Callaghan, New South Wales, Australia; 3 King's College London, National Addiction Centre, Institute of Psychiatry, London, United Kingdom

## Abstract

Jim McCambridge and colleagues reflect on how the concept of harm reduction may be being usurped by the alcohol industry.

*Please see later in the article for the Editors' Summary*

**Summary Points**

- National alcohol policies that exclude evidence-based whole-population measures because of lobbying by the alcohol industry are likely to increase rather than reduce alcohol harms.
- Corporate capture of the idea of “harm reduction” has been used by the industry to counter effective evidence-based alcohol policy development.
- The concept of alcohol harm reduction needs to be redefined to include the full range of evidence-based measures that reduce alcohol harms to public health and society.
- The ability of the alcohol industry to shape alcohol policy nationally and globally needs to be curtailed because of a fundamental conflict of interest with reducing alcohol harms.
- The WHO Global Strategy to Reduce the Harmful Use of Alcohol offers an evidence-based public health approach that can be used by national governments.

The *Alcohol Harm Reduction Strategy for England*, published a decade ago [1], was at that time criticised as being “a recipe for ineffectiveness…a textbook case of how industry interests can be brought to bear, through an ideologically friendly central government” [2]. The key criticism was that evidence-based policies of demonstrated effectiveness were ignored in favour of policies preferred by the alcohol industry [3],[4]. The resulting mix of approaches—industry self-regulation, targeting binge drinkers with largely punitive responses, public information, and school-based education—has not reduced alcohol harms. In fact, the situation has continued to worsen in England, with rates of alcohol-related hospital admissions approximately doubling within one decade [5]. Other key indicators such as liver disease death rates have also risen markedly, during a period in which they have been falling in many other western European countries [6].

The 2012 government alcohol strategy [7] for England was widely welcomed by the public health community because it announced the key policy measure of minimum unit pricing (MUP), despite otherwise having strong continuities with the ineffective approaches previously taken [8]. However, the following year saw a government U-turn on MUP [9], leaving little policy in place that can be claimed to embody meaningful strategic intent [10]. What might be termed a “lost decade” in alcohol policy in England contrasts with the situation in Scotland, where an evidence-based approach to alcohol policy has developed, with industry influence appropriately balanced [11]–[13]. Even so, the alcohol industry has successfully delayed implementation of MUP in Scotland through appeals against legal decisions within the European Union [14], mimicking the tactics of the tobacco industry [15].

## The Nature of Harm Caused by Alcohol and the Evidence on How It Should Be Reduced

Environmental factors, including those relating to history and culture, and alcohol-specific and broader social policies are responsible for both levels of drinking and the accompanying health, social, and economic harms [16]. Alcohol consumption is now understood to be a component cause of more than 200 health problems [16]. The World Health Organization (WHO) estimates that alcohol accounts for approximately 6% of all deaths, making it a leading contributor to the global burden of disease [16]. Harm increases with consumption, but the relationship varies by outcome, and with the drinker's age and sex. For example, at a population level, any consumption elevates risk for injury and hypertension, and for several common cancers [17]. For rectal cancer, risk remains low with even heavy consumption for men, but it increases exponentially with alcohol consumption for women [17]. In addition to organ damage, alcohol intoxication and dependence are the key causes of harm [18]. There is much unexplained heterogeneity in findings of health benefits of small doses of alcohol, which have attracted controversy for decades [19]–[21]. It has been suggested that there are unresolved methodological problems in observational cohort studies that have investigated these associations [22],[23], and a recent Mendelian randomisation study found no evidence of any cardiovascular benefit caused by alcohol [24]. Overall, alcohol consumption produces major harm, even taking into account any possible health benefits [16].

Risk is not confined to subgroups. Consequently, there is a need for whole-population countermeasures [25]. Systematic reviews of the effects of more than 40 types of alcohol policies and programmes consistently show that increasing price and restricting physical availability of alcohol are effective supply-side strategies for reducing alcohol-related harm [18]. On the demand side, substantially limiting the promotion of alcohol and employing strategies to reduce drunk driving (e.g., random breath testing) are effective [18]. Education and persuasion have been found to be ineffective, particularly when used in isolation, without supply-side controls [18]. Targeted harm reduction efforts have been shown to be modestly effective and cost-beneficial [26], and need to be complemented by policies that reduce harms across the population as a whole [18].

## Harm Reduction and Alcohol

The approach of harm reduction has been important in developing the science, policies, and practice of working with injection drug use at both the individual and population levels [27],[28]. The influential International Harm Reduction Association (IHRA; also more recently known as Harm Reduction International), which combines science and advocacy, officially broadened its focus in 2004 to encompass alcohol and tobacco. In seeking to challenge mainstream public health thinking that reducing population alcohol consumption is critical to reducing harm, it's advocacy of targeted interventions was appealing to the alcohol industry, and a close relationship ensued (see Box 1).

**Box 1. One Example of the Association between the Harm Reduction Movement and the Alcohol Industry**

The International Center for Alcohol Policies (ICAP) was a Washington-based organisation that was set up in 1995 by ten of the world's largest distilled spirits and beer marketers to counter the alcohol policy direction of WHO [40]. Internal documents from Phillip Morris (which then owned Miller Brewing and which retains a significant shareholding in the merged SAB-Miller [59]) revealed that they formed ICAP with the explicit objective of influencing policy [40]. ICAP is one of a range of alcohol industry bodies with which the IHRA has worked.

An ICAP and IHRA collaboration produced a book in 2007 [60] that was strongly criticised in leading specialist [43] and general medical [61] journals. One critic saw it as a disingenuous “smoke and mirrors” attempt to distract attention away from effective population-level policies [43]. Directly targeting sub-populations was presented as preferable to whole-population measures, with many of the proposed interventions lacking effectiveness data. Another critic suggested the IHRA had been “hooked in” and that it was “ill judged for the association to be linked to this lobbying exercise of the alcohol industry…. [T]he harm reduction movement needs clear blue water between itself and the alcohol industry” [61].

Critics argued that the approach advocated was likely to increase rather than reduce harm. For example, pregnant women may be targeted in order to prevent foetal alcohol spectrum disorders. Such an approach ignores the wider need to address alcohol consumption among young women (and men), which can decrease both alcohol-related pregnancies and drinking during the early stages of pregnancy, thereby reducing the prevalence of foetal alcohol spectrum disorders [43]. The alcohol industry has a vested interest in avoiding population-level policies that are expected to reduce consumption and, by extension, sales and profits.

In April 2014, the merger of ICAP with the Global Alcohol Producers Group was announced [62], and in October 2014, the name of the new organisation was presented as the International Alliance for Responsible Drinking.

The IHRA specifically promotes reducing harm by means other than reducing consumption [29]. This approach to alcohol stems from the context of illicit drugs and punitive drug policies, in which the individual's right to use drugs is defended against attempts at drug control [30]. There is a crucial ambiguity in harm reduction discourse, namely, whether the construct should be defined in terms of the methods used or the objectives or both, with the IHRA definition preoccupied with methods (not reducing consumption) at the expense of objectives [31]. There are key differences between current and earlier IHRA definitions of harm reduction [32], and one key text on alcohol harm reduction [33] notes that “most of the strategies…require a reduction in alcohol intake for their effect” [34]. Here we concentrate on harm reduction at the population level (see [35] for a guide to individual-level applications).

Mere advocacy of harm reduction, without measurement of impact, has been criticised, including by those who are sympathetic to the aims of reducing drug harms [36]. The critics are dissatisfied with the multiple meanings afforded by the concept and the lack of attention to quantifying harms [37]. There is also a tendency to conflate harm reduction with public health, rather than seeing harm reduction as one component therein, and it has been suggested that the term be abandoned in favour of describing the strategies used [38],[39]. Differentiation of harm reduction from abstinence-focused drug policy is understandable, particularly in relation to illegal drug use, where serious harms can flow from prohibition. However, it is unclear why such emphasis might be placed on distinguishing harm reduction from use reduction for alcohol, when reducing consumption so clearly reduces harm.

Such critiques [31],[32],[34],[36]–[39],[43],[61] have made no obvious dent in the position of the IHRA or in their collaboration with the alcohol industry. The most recent alcohol harm reduction conference sponsored by the International Center for Alcohol Policies (ICAP; an organisation originally formed by ten of the world's leading beer and spirits producers [40]) advocated the local responses favoured by industry in contrast to national policies that are antithetical to industry interests [41]. This orientation has also led to new “city health” conferences identifying city-level responses in the face of national policy inertia [42]. However, municipal policies should be designed to complement national public health policies, particularly as some key actions are necessarily national, for example, restricting television marketing. To present municipal and national policies as alternatives creates a false dichotomy, similar to counterposing consumption reduction and harm reduction [43].

## Corporate Capture

Corporate capture refers to the process by which corporations deliberately attempt to “dominate the information environment, so they can significantly affect decision-making” [44]. This may be achieved by managing access to, and use of, evidence by filtering it through key trusted sources such as think tanks [45] in an attempt to marginalise independent evidence. Generating doubt about the nature of the independent evidence is a key strategy of the alcohol industry and other corporate sectors, as doubts among policy-makers will restrict the actions they take [46]. Tobacco companies were the original “merchants of doubt” [47], with this strategy subsequently adopted by the ideologically motivated opposition to environmental protection. Organisations linked to the alcohol industry (including ICAP) have produced a competing alternative literature in order to undermine the use of established, independent, peer-reviewed science [40],[45]. More specific tactics used include misrepresenting unfavourable strong evidence and promoting favourable weak evidence [48]. This deliberate moulding of the evidence, or “bending science” [49], shapes ideas and influences perceptions of data by the public and policy-makers, and ideas are more important in influencing policy than evidence [50]. In this “ideas” world, the idea of harm reduction is appealing, and also attractively vague and malleable. This allows industry to claim there is disagreement within the field of public health [51], while providing a plausible rhetoric to give apparent legitimacy to resisting population-level evidence-based policies.

The libertarian strand of harm reduction thus ends up in close proximity to the neo-liberal ideas favoured by corporations, with both arguing (albeit for different reasons) that the state should not interfere in people's lives. In the “ideas” arena, effectiveness evidence is demoted to being just another consideration [43], and actions taken to promote public health are caricatured as “nanny state” paternalistic ownership of the responsibility for the population's health and welfare. This overlooks the very limited and contradictory conception of individual rights implied, and enhances the power of large corporations to shape individual behaviour to the detriment of health and wellbeing. Corporate economic imperatives extending alcohol marketing or seeking policy influence do not produce, and never will produce, true harm reduction positions. This key difference between alcohol and illegal drugs is being ignored.

## Ways Forward, Nationally and Internationally

A simplistic transfer of definitions of harm reduction from drugs to alcohol weakens society's ability to reduce the scale of the alcohol problem and to protect public health. It is thus important to expose the limitations of the alcohol-industry-favoured definition. Instead, we offer a simpler definition of harm reduction that gets to the heart of the matter. Put simply, if a policy or programme reduces harms or problems, then it is harm reduction. Evidence-based whole-population measures to reduce alcohol harm, including increasing price and reducing availability, are therefore legitimate and effective harm reduction measures within this definition. Civil society must not allow the concept of harm reduction to be defined in ways that serve corporate interests at the expense of public health.

Advocacy inspired by libertarian ideas is at odds with the evidence on how to reduce alcohol harms in the population. “Harm reduction”, as it has been applied to alcohol policy, has so far served corporate rather than public health interests. Public health approaches recognise large corporations that produce and sell drugs such as alcohol as key vectors of the global burden of disease, whose corporate social responsibility activities reflect economic rather than health or social motivations [52]. Rigorous scrutiny of the evidence on the scale and nature of alcohol harms, of the effectiveness of countermeasures, and of the behaviour of the alcohol industry is crucial. National governments need also to resist corporate efforts to subvert evidence-informed policies, in order to halt the rising levels of damage caused by alcohol to public health and society.

National alcohol policies must therefore recognise that working in “partnership” with industry has failed to reduce alcohol harm (as has been recognised in Ireland [8]); moreover, it is implausible that it ever could do so, because of an irreconcilable conflict of interest [53]. The resistance of the alcohol industry, or, indeed, any other rational economic actor whose interests are threatened, is understandable, and the means used to influence policy are deserving of in-depth investigation. The alcohol industry's favoured definition of harm reduction actually entails harm promotion, however well-constructed the smokescreen of self-serving ideas. The basis for action by national governments to regulate the industry in the public interest is now available; alcohol harm reduction will be best achieved by reducing overall consumption through increased price and reduced availability and marketing, as the international peer-reviewed evidence makes abundantly clear [18]. This means the population, as a whole, drinking less.

National alcohol policies also have global contexts [12],[54],[55]. The alcohol industry is aggressively expanding in low- and middle-income countries and seeking to influence national alcohol policies in so doing [56]. The WHO Global Strategy to Reduce the Harmful Use of Alcohol [57] offers a broad-based public health strategic approach with ten “target areas” for national policy development. It is entirely compatible with our proposed definition of harm reduction, and includes as one target area “reducing the harm from alcohol intoxication and drinking without necessarily affecting the underlying alcohol consumption…within a broader strategy that prevents or reduces the negative consequences of drinking and alcohol intoxication.” [57] All nine other target areas operate by reducing consumption via supply- or demand-side mechanisms (e.g., higher taxes and alcohol advertising bans). WHO thus regards measures to make public drinking contexts safer, for example, as just one element of a broader public health strategy.

Like corporations in other areas, the alcohol industry claims a role in policy-making at the national level in order to create regulatory environments conducive to corporate interests [49]. The editors of the leading journal *Addiction* have observed: “It may take decades to reverse the epidemics of alcohol abuse that emerge when industry-favourable policies trump public health initiatives” [54]. Margaret Chan, Director-General of WHO, recently felt it necessary to restate: “In the view of WHO, the alcohol industry has no role in the formulation of alcohol policies, which must be protected from distortion by commercial or vested interests” [58]. The stakes are high, and there should be no scope for ambiguity in alcohol policy about the role of the industry.

## Abbreviations

ICAP: International Center for Alcohol Policies
IHRA: International Harm Reduction Association
MUP: minimum unit pricing
WHO: World Health Organization
